# Motion Optimization and Control of Single and Multiple Autonomous Aerial, Land, and Marine Robots

**DOI:** 10.3390/s23010087

**Published:** 2022-12-22

**Authors:** Reza Ghabcheloo, António Pascoal

**Affiliations:** 1 Faculty of Engineering and Natural Sciences, Tampere University, P.O. Box 1001, 33014 Tampere, Finland; 2 Institute for Systems and Robotics (ISR), Instituto Superior Tecnico (IST), Torre Norte, Piso 8, Av. Rovisco Pais, 1, 1049-001 Lisbon, Portugal

*You always admire what you really don’t understand* (Blaise Pascal)

Fast-paced developments in the fields of aerial, land, and marine robotics are steadily paving the way for a wide spectrum of scientific and commercial applications of autonomous vehicles with far-reaching societal implications. Autonomous robots equipped with advanced sensors and manipulators afford humans the capability to operate seamlessly in remote and hazardous environments, as if they were extensions of our eyes and hands. Robots have become the tools par excellence for scientists and commercial operators to explore and monitor the state of heterogenous environments on Earth, inspect offshore wave and energy infrastructures, monitor the growth of crops, and transport goods, among a myriad of other activities. Groups of robots acting in cooperation have started to impact the development of multiple system platforms for adaptive environmental sampling, search and rescue operations in hard-to-access regions, and even coordinated image-taking in the movie and sports industries. The types of robots used are highly heterogeneous and cater to specific user-defined requirements for operations in the air, on land, and at sea. Notwithstanding this diversity, they have in common a number of attributes that are key to their capability to explore or act upon the environment with great agility while exhibiting high levels of performance, resilience, adaptability, and safety.

It is against this backdrop of ideas that this reprint addresses fundamental problems that are at the root of the development of a new breed of heterogenous robots that can act in isolation or cooperatively towards the execution of a wide spectrum of mission scenarios. Representative examples include the study of single and cooperative motion-planning methods with a view to meeting temporal and energy constraints in the presence of robot dynamic constraints, while taking explicitly into account the topology of the underlying communication networks and inter-vehicle and vehicle–obstacle avoidance requisites; the creation of safe and emergent behaviors in a distributed manner, at both the motion planning and control levels; the incorporation of event-driven communication strategies to try and reduce the amount of information exchanged among the different agents; the study of new methods to solve constrained optimal control problems efficiently in a receding horizon fashion; the development of effective techniques for adaptive and robust control in the presence of plant model uncertainty of partially known models, especially for safety critical systems; and the study of how advanced perception can be brought to bear on the reformulation of the above problems in a sensor-based context, yielding challenging questions in the area of visual and acoustic-based servoing, object tracking, and obstacle detection and avoidance. The reprint includes a number of chapters that focus on theoretical and practical issues pertaining to motion optimization and control, with special emphasis on, but not limited to, single and multiple autonomous aerial, land, and marine robots.

Kielas-Jensen et al. [1] present a method for the generation of trajectories for autonomous vehicles that exploits the use of Bernstein polynomial approximations to transcribe infinite-dimensional optimization problems into nonlinear programming problems. These, in turn, can be solved efficiently using off-the-shelf optimization solvers. The main motivation for this approach stems from the fact that Bernstein polynomials possess favorable geometric properties and yield computationally efficient algorithms that enable a trajectory planner to efficiently evaluate and enforce constraints along the vehicles’ trajectories, including maximum speed and angular rates as well as the minimum distance between trajectories and between the vehicles and obstacles. To support the application of the method described, an open-source toolbox called BeBOT (Bernstein/Bézier Optimal Trajectories) is introduced that implements key operations and algorithms involving Bernstein polynomials. The toolbox can be used to efficiently generate feasible and collision-free trajectories for single and multiple vehicles.

Sabetghadam et al. [2] introduce a distributed algorithm to generate collision-free trajectories for a group of quadrotors flying through a common workspace. In the setup adopted, each vehicle replans its trajectory, in a receding horizon manner, by solving a small-scale optimization problem that only involves its own individual variables. A Voronoi partitioning of space is adopted to derive local constraints that guarantee collision avoidance with all neighbors for a certain time horizon. The obtained set of collision avoidance constraints explicitly take into account the vehicle’s orientation to avoid infeasibility issues caused by ignoring the quadrotor’s rotational motion. Moreover, the resulting constraints can be expressed as Bézier curves and thus can be evaluated efficiently, without discretization, to ensure that collision avoidance requirements are satisfied at any time instant, even for an extended planning horizon. The proposed approach is validated through extensive simulations with up to 100 drones. The results show that the proposed method has a higher success rate at finding collision-free trajectories for large groups of drones compared to other Voronoi diagram-based methods.

Andreasson et al. [3] describe a local planning approach for pseudo-omnidirectional vehicles, that is, vehicles that can drive sideways and rotate in place. The local planner, named MSD, is rooted in optimal control theory and relies on the formulation of a non-linear optimization problem formulation that exploits the omni-motion capabilities of the vehicle to drive the vehicle to the goal in a smooth and efficient manner while avoiding obstacles and singularities. MSDU is designed for a real platform for mobile manipulation, where one key function is the capability to drive in narrow and confined areas. Real-world evaluations show that MSDU planned paths are smoother and more accurate than those obtained with a comparable local path planner, Timed Elastic Band (TEB), with a mean (translational, angular) error for MSDU of (0.0028 m, 0.0010 rad) compared to (0.0033 m, 0.0038 rad) for TEB. MSDU also generated paths that were consistently shorter than TEB, with a mean (translational, angular) distance traveled of (0.6026 m, 1.6130 rad) for MSDU compared to (0.7346 m, 3.7598 rad) for TEB.

Srikanth et al. [4] address the problem of the fast adaptation of manipulator trajectories for task perturbations. The main objective is to deal with the fact that manipulator joint space trajectory optimization under end-effector task constraints leads to a challenging non-convex problem. Thus, a real-time adaptation of prior computed trajectories to perturbations in task constraints often becomes intractable. Existing works use the so-called warm-starting of trajectory optimization to improve computational performance. A fundamentally different approach that relies on deriving analytical gradients of the optimal solution with respect to the task constraint parameters is introduced. The proposed algorithm provides near real-time adaptation of joint trajectories for a diverse class of task perturbations, such as (i) changes in initial and final joint configurations of end-effector orientation-constrained trajectories and (ii) changes in the end-effector goal or waypoints under end-effector orientation constraints. These two examples are related to real-world applications ranging from learning from demonstration to obstacle avoidance.

Tian et al. [5] offer a solution to problems that arise in path-planning strategies that exploit the use of rapidly exploring random trees, namely long path planning time and a large number of redundant points. To this end, an improved algorithm based on a parent point priority determination strategy and a real-time optimization strategy is derived to optimize rapidly exploring random tree algorithms. First, in order to shorten the path-planning time, the parent point is determined before generating a new point, which eliminates the complicated process of traversing the random tree to search the parent point when generating a new point. Second, a real-time optimization strategy is combined, whose core idea is to compare the distance of a new point, its parent point, and two ancestor points to the target point when a new point is generated, choosing a new point that is helpful for the growth of the random tree to reduce the number of redundant points. Simulation results of a three-dimensional path planning showed that the success rate of the proposed algorithm was close to 100%. Compared with the rapidly exploring random trees algorithm, the number of points was reduced by more than 93.25%, the path planning time was reduced by more than 91.49%, and the path length was reduced by more than 7.88%. The IRB1410 manipulator was used as a test platform in a laboratory environment to assess the efficacy of the new algorithm.

Papadimitrakis et al. [6] is a contribution to the field of automatic collision avoidance for surface vessels, which has been the subject of intensive research in recent years, aiming for the development of decision support systems to aid officers in conventional vessels, or for the creation of autonomous vessel controllers. A multi-ship control problem is addressed using a model predictive controller (MPC) that makes use of obstacle ship-trajectory-prediction models that build upon the radial basis function (RBF) framework and are trained on real AIS data sourced from an open-source database. The usage of such sophisticated trajectory-prediction models enables the controller to correctly infer the existence of a collision risk and apply evasive control actions in a timely manner, thus accounting for the slow dynamics of a large vessel, such as container ships, and enhancing the cooperation between controlled vessels. The proposed method is evaluated on a real-life case from the Miami port area, and the generated trajectories are assessed in terms of safety, economy, and COLREG compliance by comparison with an identical MPC controller utilizing straight-line predictions for the obstacle vessel.

Oftadeh et al. [7] present a nonlinear and universal path-following controller for Wheeled Mobile Robots (WMRs). In contrast to the previous algorithm, the new controller solves the path-following problem for all common categories of holonomic and nonholonomic WMRs, such as omnidirectional, unicycle, car-like, and all steerable wheels. This generality is the consequence of a two-stage solution that separately tackles the platform path-following constraints and the wheels’ kinematic constraints. During the first stage, for a virtual mobile platform free from the wheels’ constraints, a strategy is developed to drive the WMR asymptotically to the desired path. The second stage accounts for the kinematic constraints imposed by the wheels. This is accomplished by casting the otherwise intractable wheels’ kinematic and nonholonomic constraints in the form of explicit proportional functions between the velocity of the platform and those of the wheels. This result leads to a closed-form trajectory generation scheme for the asymptotic path that constantly keeps the wheels’ steering and driving velocities within their pre-specified bounds. Extensive experimental results on several types of WMRs, along with simulation results for the other types, are provided to demonstrate the performance and efficacy of the method developed.

Maurya et al. [8] tackle the problem of path following of marine vehicles along straight lines in the presence of currents by resorting to an inner–outer control loop strategy, with due account for the presence of currents. The inner–outer loop control structures exhibit a fast–slow temporal scale separation that yields simple “rules of thumb” for controller tuning. Stated intuitively, the inner-loop dynamics should be much faster than those of the outer loop. Conceptually, the procedure described has three key advantages: (i) it decouples the design of the inner and outer control loops, (ii) the structure of the outer-loop controller does not require exact knowledge of the vehicle dynamics, and (iii) it affords practitioners a very convenient method to effectively implement path-following controllers on a wide range of vehicles. The path-following controller is designed at the kinematic outer loop level and issues heading commands to the inner loop. The key underlying idea is to provide a seamless implementation of path-following control algorithms on heterogeneous vehicles, which are often equipped with heading autopilots. To this end, it is assumed that the heading control system is characterized in terms of an input–output stability (IOS)-like relationship without detailed knowledge of the vehicle dynamics parameters. The stability of the combined inner–outer loops is shown formally by resorting to nonlinear control theory, wherein the cascade and feedback systems of interest are characterized in terms of their IOS properties. Tests with AUVs and one ASV in real-life conditions have shown the efficacy of the path-following control structure developed.

Jacinto et al. [9] address the problem of formation control of a quadrotor and one (or more) marine vehicles operating at the surface of the water with the end goal of encircling the boundary of a chemical spill, enabling such vehicles to carry and release chemical dispersants used during ocean cleanup missions to break up oil molecules. Firstly, the mathematical models of the Medusa class of marine robots and quadrotor aircrafts are introduced, followed by the design of single-vehicle motion controllers that allow these vehicles to follow a parameterized path individually using Lyapunov-based techniques. At the second stage, a distributed controller using event-triggered communications is introduced, enabling the vehicles to perform a cooperative path following missions according to a pre-defined geometric formation. In the next step, a real-time path-planning algorithm is developed that makes use of a camera sensor, installed onboard the quadrotor. This sensor enables the detection in the image of which pixels encode parts of a chemical spill boundary and their use to generate and update, in real time, a set of smooth B-spline-based paths for all the vehicles to follow cooperatively. The performance of the complete system is evaluated by resorting to 3-D simulation software, making it possible to visually simulate a chemical spill. Results from real water trials are also provided for parts of the system, where two Medusa vehicles are required to perform a static lawn-mowing path following the mission cooperatively at the surface of the water.

Delbene et al. [10] introduce a procedure for the autonomous landing of a quadrotor on an unmanned surface vehicle in a marine environment. The relative pose and velocity of the vehicle with respect to the quadrotor are estimated using a combination of data coming from a vision system, which recognizes a set of fiducial markers (AprilTags) located on the vehicle itself, and an ultrasonic sensor, to achieve further robustness during the final landing phase. Details on the landing strategy and on the hardware and software architectures used to implement it are provided. Software-in-the-loop tests were performed as a validation step for the proposed algorithms; to recreate realistic conditions, the movements of the landing platform have been replicated using data from a test in a real marine environment. In order to provide further proof of the reliability of the vision system, a video sequence from a manual landing of a quadrotor on the surface vehicle in a real marine environment has been processed, and the results are presented.

Walton et al. [11] address the problem of optimal defense of a high-value unit (HVU) against a large-scale swarm attack. Multiple models for intra-swarm cooperation strategies are discussed, and a framework is proposed to combine the cooperative models with HVU tracking and adversarial interaction forces. Using this setup, the problem of defending against a swarm attack is cast in the framework of optimal control under uncertain parameters. Numerical solution methods to the latter are discussed, and a consistent result for the dual problem of this framework is derived, providing a tool for verifying computational results. It is further shown that the dual conditions can be computed numerically, providing further computational utility. Finally, the numerical results are applied to derive optimal defender strategies against a 100-agent swarm attack.

Dang et al. [12] discuss important topics in the areas of over-actuated underwater robots whose actuators are propeller thrusters. In general, the positions and orientations of the latter follow classical configurations. This poses limitations on the capability of the robots and does not optimize their performance in terms of energy efficiency, reactivity, and versatility, especially when the robots operate in confined environments. In order to optimize the thruster configuration designs for underwater over-actuated systems, performance indices (manipulability, energetic, reactive, and robustness indices) are introduced. A multi-objective optimization problem is formulated and analyzed. To deal with different objectives with different units, the goal-attainment method, which can avoid the difficulty of choosing a weighting vector to obtain a good balance among these objectives, was selected to solve the problem. A solution design procedure was proposed and discussed. The efficacy efficiency of the proposed method was proven by simulations and experimental results.

Finally, Zhao et al. [13] present a tracking controller for nonlinear systems with matched uncertainties based on contraction metrics and disturbance estimation that provides exponential convergence guarantees. Within the proposed approach, a disturbance estimator is derived to estimate the pointwise value of the uncertainties, with a pre-computable estimation error bound (EEB). The estimated disturbance and the EEB are then incorporated in a robust Riemannian energy condition to compute the control law that guarantees exponential convergence of actual state trajectories to the desired ones. Simulation results on aircraft and planar quadrotor systems demonstrate the efficacy of the proposed controller, which yields better tracking performance than existing controllers for both systems.

The editors and the authors express their sincere gratitude to the publisher and members of the staff for their unwavering commitment and invaluable advice and encouragement that contributed in a very decisive manner to enriching the quality of this reprint.
To all the parents who want a future with peace for their children.Reza GhabchelooTo Stephanie, Ricardo, Ana, and Madalena, the gentle pillars of my life.António Pascoal
